# Phylogenomics of *Reichenowia parasitica*, an Alphaproteobacterial Endosymbiont of the Freshwater Leech *Placobdella parasitica*

**DOI:** 10.1371/journal.pone.0028192

**Published:** 2011-11-23

**Authors:** Sebastian Kvist, Apurva Narechania, Alejandro Oceguera-Figueroa, Bella Fuks, Mark E. Siddall

**Affiliations:** 1 Richard Gilder Graduate School, American Museum of Natural History, New York, New York, United States of America; 2 Division of Invertebrate Zoology, American Museum of Natural History, New York, New York, United States of America; 3 Sackler Institute for Comparative Genomics, American Museum of Natural History, New York, New York, United States of America; 4 Department of Biology, The Graduate Center, The City University of New York, New York, New York, United States of America; 5 Long Island University Brooklyn Campus, Brooklyn, New York, United States of America; Columbia University, United States of America

## Abstract

Although several commensal alphaproteobacteria form close relationships with plant hosts where they aid in (e.g.,) nitrogen fixation and nodulation, only a few inhabit animal hosts. Among these, *Reichenowia picta, R. ornata* and *R. parasitica*, are currently the only known mutualistic, alphaproteobacterial endosymbionts to inhabit leeches. These bacteria are harbored in the epithelial cells of the mycetomal structures of their freshwater leech hosts, *Placobdella* spp., and these structures have no other obvious function than housing bacterial symbionts. However, the function of the bacterial symbionts has remained unclear. Here, we focused both on exploring the genomic makeup of *R. parasitica* and on performing a robust phylogenetic analysis, based on more data than previous hypotheses, to test its position among related bacteria. We sequenced a combined pool of host and symbiont DNA from 36 pairs of mycetomes and performed an *in silico* separation of the different DNA pools through subtractive scaffolding. The bacterial contigs were compared to 50 annotated bacterial genomes and the genome of the freshwater leech *Helobdella robusta* using a BLASTn protocol. Further, amino acid sequences inferred from the contigs were used as queries against the 50 bacterial genomes to establish orthology. A total of 358 orthologous genes were used for the phylogenetic analyses. In part, results suggest that *R. parasitica* possesses genes coding for proteins related to nitrogen fixation, iron/vitamin B translocation and plasmid survival. Our results also indicate that *R. parasitica* interacts with its host in part by transmembrane signaling and that several of its genes show orthology across Rhizobiaceae. The phylogenetic analyses support the nesting of *R. parasitica* within the Rhizobiaceae, as sister to a group containing *Agrobacterium* and *Rhizobium* species.

## Introduction

Hematophagous leeches (Hirudinida) of the family Glossiphoniidae posses specialized organs related to the esophagous whose only known function is to house intracellular bacterial symbionts [1]–[3]. These structures, known as mycetomes or bacteriomes, show high morphological plasticity across the family ranging from granular tube-like structures circumscribing the esophagous in the genus *Placobdelloides* to distinct spheroid structures in the genus *Haementeria* [1]. In the genus *Placobdella*, the mycetomes are arranged as a pair of blind caeca about half-way down the esophagous [1], [4]. Notably, mycetomes and the associated symbionts are completely absent from those leeches in Glossiphoniidae that have given up blood-feeding entirely (e.g., species of *Glossiphonia* and *Helobdella*). Because of the retention of these organs in hematophagous glossiphoniid leeches, the bacterial symbionts likely play an important role for the hosts. It has been hypothesized that the lack of essential nutrients, such as vitamins and enzymes, brought by the leeches' restricted diet of vertebrate blood [5], is ameliorated by the provision of nutrients by bacterial symbionts housed in the mycetomes [6]. Commonly in both plants and animals, obligate bacterial symbionts (primary symbionts) are housed in a distinct set of host-cells, known as bacteriocytes, and are strongly associated with these cells, to the point that they cannot invade unspecialized tissues [7]. The importance of the leech bacterial symbionts is also suggested by their vertical transovarial transmission [4].

Although symbiotic associations between bacteria and leeches are well-documented [1], [4], [6], [8], [9], several questions concerning the details of the symbioses still remain. In particular, neither the function of the bacterial symbionts nor their putative “symbiont syndrome” has been clearly determined. The symbiont syndrome is a collective term for a set of features that are characteristic of intracellular bacterial symbionts [10], [11]. These include a reduction in genome size, A–T bias, rapid sequence evolution and frequent gene rearrangements.

Siddall et al. [4] described the alphaproteobacterium *Reichenowia parasitica* from the mycetomes of its freshwater leech host, *Placobdella parasitica*, and, hitherto, the genus *Reichenowia* (three species; *R. picta, R. ornata* and *R. parasitica*) contain the only known mutualistic, endosymbiotic Rhizobiaceae that inhabit animal hosts. Other mutualistic alphaproteobacteria inhabit plants (e.g., *Rhizobium, Agrobacterium*) and most of those that infect animals (e.g., *Brucella* spp.) are parasitic ([12]; and references therein). Among other functions, bacterial plant-symbionts aid in nitrogen fixation and nodulation in the plants, allowing for more effective nutrient uptake and rapid growth [13]. Moreover, the nitrogen fixation capability of prokaryotes has been highly studied because of its large impact on the ecosystem [14]–[16].

**Figure 1:**
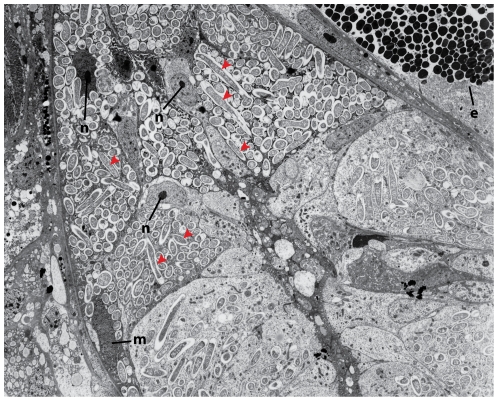
Transmission electron micrograph showing the rod-shaped morphology and several cross-sections of *Reichenowia parasitica*. The micrograph shows the inside of an epithelial cell of the mycetome from *Placobdella parasitica* at 5640x magnification, with some bacterial cells (red arrowheads), secretory esophageal cells (e), nuclei (n) and a mitochondrion (m) marked.

Using phylogenetic analyses, Siddall et al. [4] recovered *R. parasitica* within the family Rhizobiaceae but with low resolution concerning the internal placement of the species within this group. Moreover, for Siddall et al. [4], all attempts at culturing the bacteria, using various media, were unsuccessful, suggesting that the symbiont has a reciprocally obligate relationship with the host. Unculturable bacteria represent the majority of life forms [17]; many of these are endosymbionts of animal hosts and are vertically transmitted from parent to offspring, like *R. parasitica*. Taking into consideration that these bacteria prove refractory to culturing, direct and simultaneous sequencing of both associates is one of the few ways to obtain genetic material from the endosymbiont. It then becomes important to understand the diversity of the bacterial symbionts in the host. Primary evidence suggests that *R. parasitica* is the only bacterial symbiont to inhabit the mycetomal structures of the leech *Placobdella parasitica*. Several independent forms of data support this: first, multiple sequencing efforts of the mycetomes, using bacterial-specific primers for the 16S rDNA region, resulted in only a single bacterial haplotype [4]; second, fluorescent *in situ* hybridization of the mycetomes, using both alpha- and gammaproteobacterial probes, shows that only alphaproteobacteria are present and that these are found exclusively in the epithelial cell layer surrounding the sac-like structure [4] such that no contaminants would stem from intraluminal endosymbionts; third, transmission electron microscopy of the epithelial cells shows the presence of only one bacterial morphotype [4]. Interestingly, *R. parasitica* maintains a rod-shaped morphology (Figure 1), common in free-living bacteria [18]. However, a rod-shaped morphology has been described also for endosymbiotic bacteria [19], [20] and it is known that conversions from a rod shape to a sphere (but not the opposite) occur in single bacterial cultures [21], [22].

Advances in sequencing technology allow for high-throughput and high-coverage sequencing of bacterial symbionts without the need to culture the bacteria [23]. We sought to characterize and annotate a large subset of the genome of *R. parasitica* in an attempt to investigate how the symbiont may affect the host and to assess the symbiont's phylogenetic position among a wide range of bacteria, with much greater genetic coverage than that of previous phylogenetic hypotheses.

## Materials and Methods

### Leech Collection and Dissection

A total of 39 specimens of *Placobdella parasitica* were collected in Algonquin Park, Ontario, Canada in July 2009. All necessary collection permits were obtained from Ontario Parks, Canadian Ministry of Natural Resources. Most specimens were found attached to and feeding on hosts, specifically painted turtles (*Chrysemys picta*) and snapping turtles (*Chelydra serpentina*). Specimens were also collected by hand from under rocks, submerged wood and the underside of canoes. Specimens were brought back to the lab where they were dissected using a Nikon SMZ645 stereomicroscope. A total of 72 mycetomes (36 pairs) were removed from the leeches and directly transferred to Buffer AL (Qiagen Ltd.).

### DNA Extraction, Amplification and Pyrosequencing

From the mycetomes, total combined genomic DNA from both the host and the bacterial associate was extracted using DNeasy Blood and Tissue Kit (Qiagen Ltd.) following the manufacturer's protocol with the addition of 1 µl of ribonuclease in order to increase the DNA/RNA ratio (i.e., 260/280 ratio). Due to the high amount of DNA required for pyrosequencing (10 µg), the extracted DNA was subjected to whole-genome amplification using REPLI-G UltraFast Mini Kit (Qiagen Ltd.). The amount of DNA was calculated by fluorometry to be in excess of 10 µg using Quant-iT PicoGreen Kit (Invitrogen). A GS Titanium Shotgun sequence library was prepared and massively parallel pyrosequencing of the amplicon was performed on the GS/FLX Titanium Shotgun XLR sequencing platform at SUNY Buffalo's Center for Excellence in Bioinformatics and Life Sciences (Buffalo, New York).

### Assembly, Subtractive Scaffolding and Orthologue Recovery

The combined pool of host and symbiont DNA fragments from the FLX run were jointly assembled into contigs using Newbler ver. 2.3 (454 Life Sciences) and employing the “-large” option.

To separate the host and symbiont DNA, contigs were subjected to subtractive scaffolding: they were used as queries against 40 selected alphaproteobacterial target genomes and 10 non-alphaproteobacterial genomes (Beta-, Gamma-, Delta-, and Epsilonproteobacteria, as well as Firmicutes, Aquificae, Bacteroidetes and Cyanobacteria), both from endosymbiotic and free-living bacteria, and with largely varying genome sizes (Table S1). Alphaproteobacteria were over-represented because of previous phylogenetic hypotheses placing *R. parasitica* within this class [4], [6]. Moreover, the contigs were queried against the only sequenced leech genome, *Helobdella robusta* (family Glossiophoniidae), which coincidentally is in the same taxonomic family as *Placobdella parasitica* [24], [25]. The leech genome is available at the DOE Joint Genome Institute portal website (http://genome.jgi-psf.org/Helro1/Helro1.home.html). Two local searches were performed using the BLASTn protocol applying default settings, one with a cut-off expectation value of 1E^−5^ and the other with 1E^−2^. All contigs simultaneously matching both associates using the 1E^−2^ cut-off rate were also deleted from the 1E^−5^ data set. The criteria were asymmetric in order to enrich for bacterial sequences in our retained DNA-pool; the purpose being to completely purge the leech DNA from the data set, including putative chimeric sequences resulting from the nested assembly of both associates. With these criteria, each of the retained hits necessarily had a three orders of magnitude lower e-value when queried against bacteria than when queried against leech. Annotations of the *R. parasitica* sequences follow the GenBank annotations of the 50 bacterial genomes and inferences of molecular function follow information from UniProt and appropriate references.

Retained bacterial contigs also were subjected to gene prediction using GeneMark ver. 2.4 [26], which employs both ORF's (Open Reading Frames) and hidden Markov models for prediction, and using *Sinorhizobium meliloti* as a scaffold genome. This species was chosen by virtue of previous phylogenetic hypotheses showing a close relationship between *R. parasitica* and *S. meliloti* [6]. Resulting nucleotide sequences of putative genes were translated into stop-codon-free amino acid sequences by GeneMark and these were then queried against the 50 bacterial proteomes downloaded from GenBank. Orthologues were recovered employing a tree-based approach as implemented in OrthologID [27]. A 70% similarity cut-off rate and a lower e-value limit of 1E^−10^ were employed. OrthologID was also used to align the amino acid sequences using multiple sets of alignment parameters and employing the MAFFT L-INS-i algorithm [28].

### Clusters of Orthologous Groups (COG's)

The predicted *Reichenowia parasitica* genes as well as genes from species of *Agrobacterium*, *Mesorhizobium*, *Wigglesworthia*, *Buchnera* and *Escherichia* each were compared to the NCBI COG database (http://www.ncbi.nlm.nih.gov/COG/) by in-house scripting. The species were chosen with respect to their phylogenetic placement and life history strategies (see Results). A *ruby* script was run locally to compare each of the genes against the database and only the best hit for each gene was retained.

### Phylogenetic Analyses

The matrix of the aligned amino acid orthologues recovered by OrthologID was subjected to parsimony analysis using TNT [29] and likelihood analysis using RAxML ver. 7.2.8 [30]. In TNT, a New Technology search was conducted employing sectorial searching, with the tree fusing and ratcheting algorithms turned on. Trees were retrieved by a driven search using 100 initial addition sequences and requiring that the minimum length tree be found a total of 10 times. All characters were equally weighted and non-additive, and gaps were treated as missing data. Support values for nodes were also calculated in TNT through both standard bootstrap resampling and partition bootstrapping [31] using the *blockboot.run* script available on the TNT Wiki site (http://tnt.insectmuseum.org/index.php/Manual) for the latter. Both bootstrap analyses employed 100 iterations, each subjected to ten iterations of ratcheting and three rounds of tree fusing after an initial five rounds of Wagner tree building. To examine the relative support of each separate locus predicted by GeneMark for the tree obtained from all of the data, constrained analyses were employed in PAUP* ver. 4.0b10 [32].

For the likelihood analyses, a heuristic search was performed under both PROTCATJTTF and PROTGAMMAJTTF models of protein evolution, treating the blocks as a single set. Runs were performed for 100 iterations with an initial 25 CAT rate categories and final optimization with 4 gamma shape categories. Bootstrap analysis employed the PROTGAMMAJTTF model for 100 pseudoreplicates with a random starting-tree for each replicate.

The outgroup taxa were chosen to accommodate all of the proteobacterial classes, as well as several other classes while, at the same time, including only taxa for which there are entire genomes already sequenced. The trees were rooted with *Aquifex aeolicus* (Aquificae), following the hypotheses of Snel et al. [33].

## Results

### Sequence Analysis

**Figure 2:**
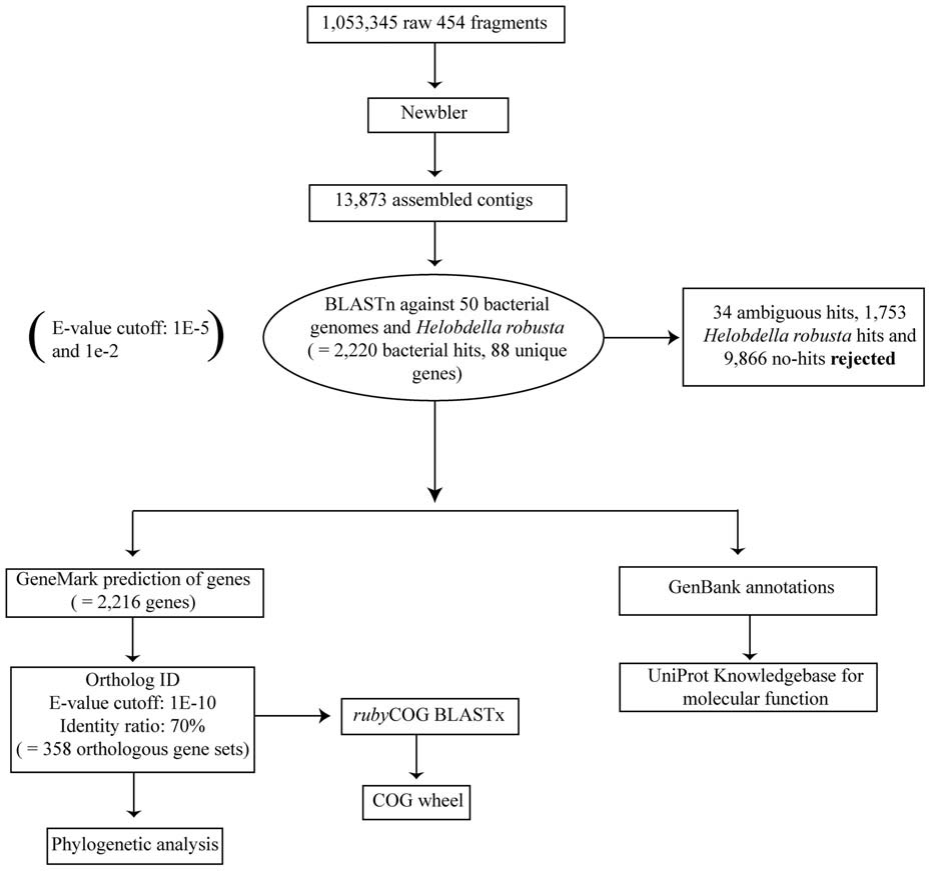
Main workflow followed in this study.

The main workflow of this study is presented in Figure 2. The pyrosequencing returned 1,053,345 fragments of mixed host and symbiont DNA (GenBank Sequence Read Archive [SRA] accession number SRA030522.3) and these were assembled into 13,873 contigs by Newbler. The BLASTn search using a cutoff e-value of 1E^-5^ resulted in 2,247 of the contigs hitting bacteria alone, 1,753 contigs hitting leech alone, seven contigs hitting both the 50 bacterial genomes and the leech and 9,866 contigs not hitting either of these (Table 1). Among the seven ambiguous contigs, four hit bacteria with very low e-values (1E^−37^–1E^−175^) while, at the same time, showing high e-values for the leech hit (1E^−6^–10^−10^). The remaining three hits showed the reverse scenario with low e-values for leech hits and high e-values for bacterial hits, implying that these seven contigs are not shared by the leech and bacterial genomes but, rather, are artifacts of the protocol used for the BLAST search. The second BLASTn search (1E^−2^) resulted in 2,611 of the contigs hitting bacteria alone, 4,553 contigs hitting leech alone, 207 contigs hitting both bacteria and leech and 6,502 contigs hitting neither bacteria nor leech (Table 1). From the resulting 2,247 contigs matching bacteria at 1E^−5^, 27 out of the total 207 contigs matching both associates at 1E^−2^ were removed. The remaining 180 ambiguous contigs were predicted leech hits at 1E^−5^ and also hit bacteria with marginal e-values at 1E^−2^; these were already removed from the data set after the 1E^−5^ search. After pruning, 2,220 definitive bacterial contigs were retained. Descriptions of all hits with hit counts are presented in Table S2. The 2,220 contigs, in turn, pertained to 88 uniquely annotated genes among the 50 bacterial genomes and 39 of these were hit with a perfect e-vaule (0). As was expected, most of the bacterial contigs hit multiple times for the same annotated locus but with differing e-values and starting/stopping points for a total of 42,025 hits stemming from the 2,220 *R. parasitica* contigs. The most frequently found annotations of the *R. parasitica* contigs, in terms of representation, seem to relate to two biological processes: transportation and catalytic activity of various components. Other rather highly represented biological processes among the contig matches were DNA transcription and metabolic processes, and for several of the hit-descriptions of our contig matches there is little or no information in the Protein Knowledgebase, UniProtKB (e.g., polyhydroxyalkonate synthesis repressor; 1975 hits).

**Table 1:** Distribution of leech and bacterial BLASTn hits among the 13,873 contigs assembled from the 454 pyrosequencing reads.

Cutoff e-value	# Bacterial hits	# Leech hits	# Ambiguous hits	# No match hits
1E^−5^	2,247	1,753	7	9,866
1E^−2^	2,611	4,553	207	6,502

Ambiguous hits indicate those contigs that matched both leech and bacteria simultaneously. In the 1E^−5^ protocol, both the leech and ambiguous hits, as well as the contigs without match were deleted from the data set. Also, 27 out of the 207 contigs matching both associates at 1E^−2^ (the remaining 180 hits were predicted leech hits at 1E^−5^) were deleted from the 2,247 contigs matching bacteria at 1E^−5^ resulting in 2,220 definitively bacterial hits in the data set. See the results section for further discussion.

### Gene Prediction and Phylogeny

Among the 2,220 *R. parasitica* contigs, GeneMark predicted 2,916 genes for a total of 1,785,377 basepairs. The G+C content pertaining to these was 62.78%. OrthologID identified a total of 9,135 orthologous genes among the 51 (including *R. parasitica*) genomes, 358 of which included an *R. parasitica* orthologue (3.9% of the total gene-groups). That is, among the 2,916 *R. parasitica* genes predicted, 358 were found orthologous to any of the genes in the 50 bacterial genomes. These orthologues accounted for 181,848 aligned amino acids sites, and these were jointly submitted to TNT and RAxML for phylogenetic analyses. The percentage of missing data amounted to ∼55% within the total data set, due to numerous instances of gene loss, common in bacterial genomes and anticipated to be even more so in endosymbionts [34], [35].

**Figure 3:**
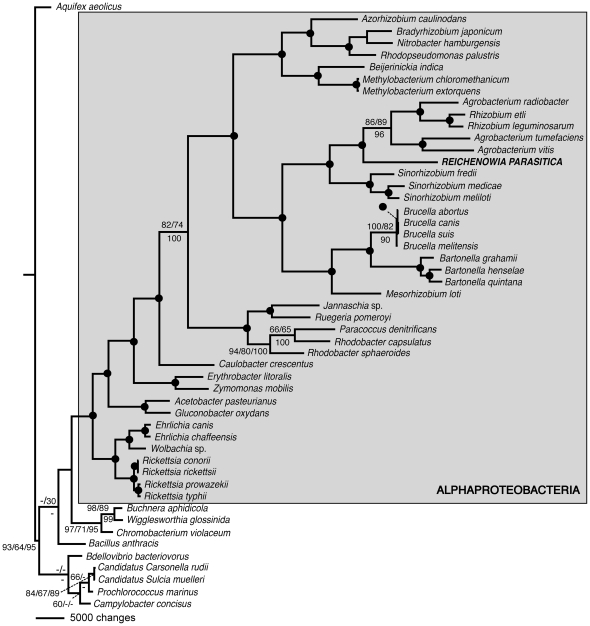
Single most parsimonious tree (length = 408,192 steps, consistency index = 0.647 and retention index = 0.648) recovered from the phylogenetic analysis of the 358 orthologues across 51 taxa. The topology is identical to the maximum likelihood tree recovered by RAxML. Values above the nodes are standard bootstrap re-sampling and partition bootstrap values, respectively, and below the nodes are likelihood bootstrap values. Solid black circles denote nodes with bootstrap support ≥90% for all three support measures.

Out of the 181,848 aligned amino acid sites, 58,887 were parsimony informative. Each of the retained gene groups containing an *R. parasitica* orthologue (n = 358) was used as an independent block both for the partition bootstrapping and the partition congruence test. The TNT run and both RAxML runs (using PROTCATJTTF and PROTGAMMAJTTF models of evolution) returned optimal trees with identical topologies; a single most parsimonious tree with a length of 408,192 steps for the TNT run and a tree with an ln *L* of −2,262,856.651 for the RAxML run using the PROTGAMMAJTTF model. In the tree (Figure 3), the alphaproteobacteria, as well as each of the families contained therein were recovered as monophyletic, and 33 out of the 48 nodes show high support for all three support measures (>90% parsimony bootstrap support: bs; parsimony partitioned bootstrap support: pbs; likelihood bootstrap support: lbs). *Reichenowia parasitica* was recovered nested within the Rhizobiaceae (100% bs; 100% pbs; 100% lbs), as sister to a monophyletic cluster consisting of *Agrobacterium* and *Rhizobium* species (86% bs; 89% pbs; 96% lbs), and this group in turn placed as sister to the *Sinorhizobium* species (100%bs; 100% pbs; 100% lbs). Rhizobiaceae (the genera mentioned above) was recovered as sister to a larger assemblage containing species of the families Brucellaceae, Bartonellaceae and Phyllobacteriaceae (100% bs; 97% pbs; 100% lbs). In addition, relative support conferred by each locus (n = 358), for the placement of *R. parasitica* within Rhizobiaceae was assessed by employing constraint trees in PAUP* (under the parsimony criterion). That is, for each locus, two values were found: one constraining to include *R. parasitica* in Rhizobiaceae, and another excluding it from Rhizobiaceae (but imposing no other relationship constraints on taxa). In the combined analysis, the number of extra steps incurred by combining the partitions was 9,867 and the difference in length between the best trees constraining *R. parasitica* to be inside and outside of Rhizobiaceae was 232 steps (∼2.4% of the total incongruence). A total of 206 loci (58%) support the placement of *R. parasitica* inside of Rhizobiaceae, whereas only 45 partitions (13%) do not support its placement inside the family. The sum of the number of extra steps from partitions that do not support *R. parasitica* inside of Rhizobiaceae is 371. However, 1057 extra steps are required to remove *R. parasitica* from the family. In other words, there is almost three times as much information supporting the placement of *R. parasitica* inside of Rhizobiaceae, as opposed to outside the family. Though none of the 45 partitions individually place *R. parasitica* in Rhizobiaceae, even combining these 45 loci again places the species inside of the family.

### COG Analyses

**Figure 4:**
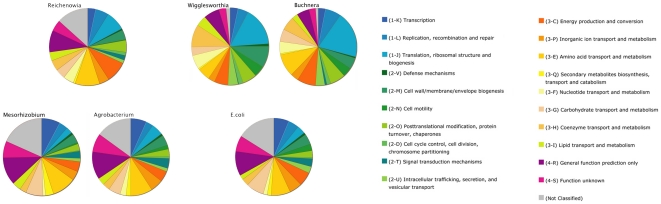
Comparison of Clusters of Orthologous Groups (COG's) between animal and non-animal endosymbionts. The 358 *R. parasitica* orthologues, as well as the genomes of species of *Agrobacterium, Mesorhizobium, Wigglesworthia, Buchnera* and *Escherichia* were used as queries against the database. The different colors denote separate functional groups to which the genes are linked. In both of the phylogenetically related groups (left: *Reichenowia, Agrobacterium* and *Mesorhizobium*, and right: *Wigglesworthia, Buchnera* and *Escherichia*) the topmost wheels represents animal-inhabiting endosymbionts, whereas the bottommost wheels represent non-animal endosymbionts. When compared to the non-animal endosymbionts, the animal endosymbionts each show a decrease in the proportion of genes related to 1-K (transcription), and an increase in the proportion of genes related to 1-J (translation, ribosomal structure and biogenesis), 2-O (posttranslational modification, protein turnover, chaperones), and 3-F (nucleotide transport and metabolism).

Alternative to the annotations of the contig matches mentioned above, using the amino acids of the predicted genes (employing the 358 partitions as separate loci) as queries against the COG database unveiled three main functional groups to which the *R. parasitica* genes seem to be related. These were: (i) information storage and processing, (ii) cellular processes and signaling and (iii) metabolism. The distributions and subdivisions of these COG-groups are presented in Figure 4. The *R. parasitica* COG-groups were also compared to those of the closely related plant-inhabiting *Agrobacterium* and *Mesorhizobium* to investigate for patterns in the devotion of the genome to particular processes as a result of a change in endosymbiotic lifestyle from a plant to an animal host. To corroborate the findings, the COG's of *Wigglesworthia* and *Buchnera*, both gammaproteobacterial endosymbionts of animals, were compared to the ubiquitous gammaproteobacterium, *Escherichia coli*. The patterns within and across these largely separate phylogenetic clusters were then investigated. When compared to related non-animal endosymbionts, *Reichenowia, Wigglesworthia* and *Buchnera* all show a decrease in the proportion of genes devoted to transcriptional processes (Figure 4; dark blue field 1-K). Furthermore, they all show an increase in proportional gene-devotion to each of translation, ribosomal structure and biogenesis (Figure 4; light blue field 1-J), posttranslational modification, protein turnover and chaperones (Figure 4; light green field 2-O), and nucleotide transport and metabolism (Figure 4; light yellow field 3-F).

## Discussion

Beyond corroborating and solidifying the hypothesis that *Reichenowia parasitica*, a mutualistic, intracellular bacterial symbiont of the fresh-water leech *Placobdella parasitica*, places phylogenetically among the alphaproteobacterial Rhizobiaceae, the present study also reveals several interesting features of the genomic makeup of the bacterium. Some of the BLAST-based hits, e.g., histidine ammonia-lyase (1100 hits among the *R. parasitica* contigs; Table S2) are fairly common across prokaryotes and eukaryotes alike [36] while other loci are more elusive, making them of special interest based on our, albeit limited, knowledge of the biology of the symbiont. Some of these loci are discussed below (see Table S2 for the full list of hits) and a broad phylogenetic discussion is presented. Insofar as the *R. parasitica* genome was only partially sequenced, no examination of the functional consequences of the lack of genes can be definitively performed.

### Cation Pump Membrane Proteins (Nitrogen Fixation)

Because of the close relationship of *R. parasitica* to each of *Rhizobium* and *Sinorhizobium*, it is likely that these taxa share genes by virtue of having a rather recent common ancestor. Both of the mentioned genera have been frequently studied for their established symbiosis with legumes, and in particular for their nitrogen fixation capabilities [37]. Already, Siddall et al. [4] noted that *Reichenowia* species are especially interesting because of their putative role in nitrogen metabolism in the leech hosts. Here, we identified 34 contigs that show high sequence similarity to the cation pump membrane proteins of *Rhizobium etli*, and 6 contigs that show similarity to potassium ion transmembrane transporter proteins from *Sinorhizobium medicae* (Table S2). Cation pump membrane proteins, such as FixG, FixH, FixI or Na^+^/K^+^ ATPase, are required for symbiotic nitrogen fixation and it has been hypothesized that these genes are private (i.e., present only in a specific group, but not necessarily in all members of that group) to symbiotic bacteria, as they do not hybridize well with DNA from free-living bacteria [38]–[40]. Notwithstanding the K^+^ ion transporters, it is unclear which type of cation pump membrane protein the *R. parasitica* contigs are related to but, regardless, they may be involved in nitrogen metabolism in the host. In addition, cation pumps have been shown to be coupled with redox processes [38], [41] and numerous *R. parasitica* contigs show sequence similarity to known oxyreductase proteins (e.g., NuoK2 NADH: quinone oxidoreductase in *Sinorhizobium meliloti*, XoxF in *Methylobacterium extorquens* and oxidoreductase in *Agrobacterium vitis*; see Table S2), providing a possibility for coupling of cation pumps and redox systems in the bacteria.

Nitrogen fixation is vital for biosynthesis of amino acids in plants and has been coupled with metabolic processes in animals. For example, in the shipworm *Lyrodus pedicellatus* (Bivalvia), nitrogen fixation by the endosymbiotic gammaproteobacteria *Teredinibacter turnerae* has enabled the shipworm to survive and grow on a nitrogen-poor diet [42]. That is, the *L. pedicellatus* - *T. turnerae* system is an example of a symbiosis, in which atmospheric nitrogen is converted into animal biomass. To this end, the leech host, *Placobdella parasitica*, may increase its growth due to the increase in organic nitrogen provided by the bacteria. Moreover, the leech may be alleviated from costly inorganic nitrogen excretion due to the conversion of inorganic to organic nitrogen by the bacteria.

### Iron Siderophore/Cobalamin (Vitamin B_12_) ABC Transporters

ATP binding cassette (ABC) transmembrane transporters consist of two membrane-spanning domains, which form a translocation pathway, and two cytoplasmic ABC domains, which power the transport process [43]. In prokaryotes, ABC transporters are chiefly devoted to the export and import of essential nutrients, such as iron and vitamin B_12_ in *E. coli* [44]. Several nutrients, including vitamin B_12_, are low in vertebrate blood such that hematophagous parasites must rely on a symbiotic organism that has the capability of synthesizing and transporting them to the host [45]. Dietary supplementation experiments have shown that endosymbiotic bacteria (*Wigglesworthia*) in the bloodfeeding tsetse fly play a role in vitamin B metabolism [5], [46]. The primary diet for *Placobdella parasitica* is poikilothermic vertebrate blood, which is low on vitamin B_12_. Therefore, it would make sense for the leech to harbor bacterial symbionts with the capacity for synthesizing and transporting vitamin B_12_ across cell membranes to host receptors. An iron siderophore/cobalamin (vitamin B_12_) ABC transporter from *Rhodobacter capsulatus* significantly matched 19 contigs in *R. parasitica*, putatively indicating that, as speculated by Perkins et al. [6], the bacteria supply essential nutrients to the leech host.

### Prevent-Host-Death (phd) Family Proteins

*Escherichia coli* is the most well known symbiont to exhibit plasmid addiction. Plasmid-encoded addiction genes are thought to be involved in conserving low-copy bacterial plasmids by selectively killing cells that have lost a plasmid. For the prevent-host-death system, this entails two genetic markers: the toxin (death-on-curing; *doc*) and the antitoxin (*phd*). Functionally, in cells that posses the focal low-copy plasmid, *phd* must be maintained at a sufficient level to inhibit the function and/or synthesis of the toxin, allowing survival of plasmid-possessing cell-lines and ultimately the plasmids themselves [47]. Because of the high energy-expenditure involved in producing antitoxins by the cells only to maintain status quo, plasmid addiction has been referred to as a Red Queen-type system. In total, 26 *R. parasitica* contigs were matched with DNA sequences annotated as *phd*-type proteins from *Methylobacterium chloromethanicum* (alphaproteobacteria) (Table S2). As our knowledge of the plasmid set-up for *R. parasitica* is virtually non-existent, this finding at least indicates that the bacteria posses plasmids (although some bacterial toxin-antitoxin systems are chromosomally encoded; e.g., [48]). A more in-depth study of the plasmid addiction associates would be beneficial as it would allow for an understanding of the plasmid count, composition and expression levels in the bacterial symbiont, as well as the underlying survival techniques of the plasmids.

### Antirestriction Family Proteins

Antirestriction family proteins are commonly involved in overcoming restriction barriers during establishment after conjugative transfer. For example, in *E. coli* antirestriction proteins of type Ard (Alleviation of Restriction of DNA) specifically affect the restriction activity of type I restriction-modification systems, and may be involved in the regulation of gene transfer between bacterial genomes [49]. Moreover, the restriction-modification system is important in limiting the transfer of genetic elements responsible for bacterial resistance to antibiotics [50], making the inhibition of this system by the antirestriction proteins of human concern.

The BLASTn search, performed here, recovered 125 *R. parasitica* contigs with low e-values when compared to genes annotated for antirestriction family proteins (Table S2). As with the *phd* family proteins (see above), this result indicates that *R. parasitica* does possess plasmids, unlike several other bacterial symbionts [51]. In regards to the function, it is still unclear if *R. parasitica* uses the putative genes for any of the reasons mentioned above. When compared to the protein sequence of annotated antirestriction proteins from *Agrobacterium vitis* (GenBank Protein ID: YP_002551430.1), one of the contigs shows 27% conservation (for shared amino acid positions). At this stage, we cannot conclude that the putative antirestriction protein present in *R. parasitica* does not function in the same way as in other prokaryotes, as a counteract against the restriction-modification system ultimately allowing foreign DNA to enter the cell. However, without performing functional analyses (such as mutagenesis), it would be premature to infer that these proteins are functionally related.

### Autoaggregation Proteins

Autoaggregation proteins share homology with adhering proteins of e.g., *Rhizobium* species [52]. Adhering proteins are calcium-binding proteins that recognize receptors on the bacterial surface, leading to congregation of cells. In plant associated symbionts, it is thought that the proteins are involved in the attachment process to plant lectins [53]. For many animal pathogens (e.g., *Bartonella* spp.), an important factor for virulence is that the bacteria can adhere to the host-cell surface or the extracellular matrix components. It is likely that *R. parasitica* uses these putative adhesion proteins in much the same way. By sticking to the mycetomal cell walls, and to each other, the bacteria can maintain their position in the cell. In fact, transmission electron microscopy has shown that the cytoplasmic space of epithelial cells in the mycetomes of *Placobdella* species are almost completely filled with bacteria [4], suggesting the need for adhesion to the host-cell walls. A total of 1972 *R. parasitica* contigs hit autoaggregation protein (adhering protein from *Rhizobium etli* CFN 42) with significant e-values (Table S2).

### Phylogeny

Based on both parsimony and likelihood algorithms, Siddall et al. [4] performed a phylogenetic analysis of three *Reichenowia* species using 16S and 23S ribosomal RNA. That study, corroborated by the present study (see Figure 3), recovered *R. parasitica* among the Rhizobiaceae as sister to a group including the *Rhizobium* and *Agrobacterium* species. Later, Perkins et al. [6] recovered the same three species as sister to a group containing *Sinorhizobium meliloti* (with an unresolved position), *Brucella melitensis* and *Brucella henselae*. In the analysis performed by Perkins et al. [6], the *Agrobacterium* species and the *Rhizobium* species were recovered as consecutive sister-groups to this larger group. From a biological standpoint, and because contemporary bacterial taxonomy and phylogenetics focuses largely on 16S and 23S rDNA [54]–[56], it is comforting to know that the phylogenetic signal present in 16S or 23S alone is rather concordant with that of the 358 orthologues used here.

The well-supported plant-symbiont affiliation of *R. parasitica* raises some interesting questions concerning the evolutionary history of the bacteria. Because of the basal position of the *Sinorhizobium* species in the phylogenetic hypothesis presented here, the ancestral life history trait of the Rhizobiaceae seems to be plant symbiosis, with *R. parasitica* showing a host switch from plant to leech. This is further supported by the finding of several plant-associated genes, such as phosphatase, in the genome of *R. parasitica* (Table S2). Out of the 358 orthologues detected among the *R. parasitica* contigs, several were private to *Rhizobium, Agrobacterium, Sinorhizobium* and *Reichenowia*, possibly indicating common ancestry among these genera. However, it is also possible that the ancestor of the *R. parasitica* was free-living by virtue of the rod-shape of the bacterium, a shape common in several other free-living taxa [57], and it is possible that the same free-living ancestor also evolved into the plant-symbiotic bacteria that we see today. A more taxonomically rich study of the alphaproteobacteria as a whole will likely shed light on the ancestral life-history strategy of the Rhizobiaceae.

**Figure 5:**
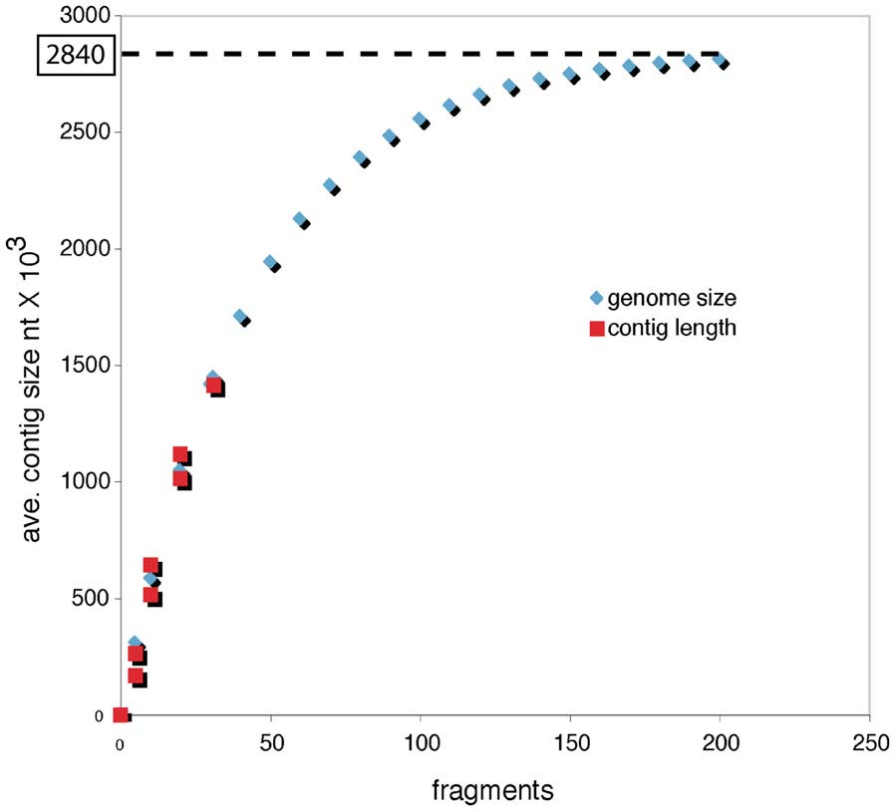
Estimation of the genome size of *Reichenowia parasitica* based on Newton-Rhapson estimation on a non-linear general logistic equation. Blue diamonds denote the general logistic equation with the asymptotic end-point being predictive of full genome size. Red squares denote the average contig size at 16.5%, 33%, 66% and 100% of the total bacterial pyrosequencing fragment pool, respectively. The estimated end-point and thus the full genome size is predicted at 2.84 Mbp.

The phylogenetic hypothesis also enables some inferences regarding the currently unknown genome size of *R. parasitica*. Among other things, an understanding of the genome size of the symbiont may guide future sequencing efforts of its entire genome. The size of the chromosomal genomes of the *Agrobacterium* and *Rhizobium* species (sister to *Reichenowia*) used here range between 5.66–7.42 megabasepairs (Mbp), whereas the *Sinorhizobium* species (basal to *Reichenowia*) possess chromosomal genomes in the range of 6.71–6.89 Mbp. By extension, it is probable that the genome size of *R. parasitica* is somewhere in the vicinity of that of its closest relatives, between 5.66–7.42 Mbp. However, we also performed a genome-size calculation based on statistical inferences. We examined the trend using average, not total, contig length (fragments assembled using EGassembler [58]) for 16.5%, 33%, 66% and 100% of the total bacterial pyrosequencing fragment pool with the asymptotic end-point being predictive of full-genome size using Newton-Rhapson estimation on a non-linear general logistic equation [GENOME*(1-(1/e^(obs*CONSTANT)^))]. The resulting predicted genome size of *R. parasitica* was 2.84 Mbp (Figure 5). This value corresponds with the reduced genomes evident in several other animal endosymbionts and would imply that *R. parasitica* displays at least one feature of the symbiont syndrome.

Sequencing the entire genome of *R. parasitica* should be the focus of future studies as it would also allow for insights into the full genomic makeup of the symbiont, including the functional consequences of the absence of genes, and the potential finding of more genes related to the endosymbiotic lifestyle of this non-parasitic, animal-inhabiting alphaproteobacterium.

There are, of course, numerous ways of assembling and managing short sequence reads. Although the methods and results conveyed here are straight-forward, only a small subset of the bacterial contigs (n = 358) were analyzed. We are currently exploring different, and possibly more efficient, ways of assembling the fragments and analyzing the data, chiefly to identify the origin of the 9,866 contigs that did not have a match. However, it is our hope that the preliminary data shown here will serve as a stepping-stone for future studies of this intriguing symbiosis.

## Supporting Information

Table S1 **List of species used for subtractive scaffolding, orthologue recovery and phylogenetic analysis.** Bold font denotes the non-alphaproteobacterial species. GenBank RefSeq refers to the submission inclusive of the entire genome.
(DOC)

Table S2 **Description of BLASTn Hits Encountered using the** ***Reichenowia parasitica*** **Contigs as Queries Against 50 Selected Bacterial Genomes.** All hits matched at 1E^-5^ or lower. Hit descriptions follow the GenBank annotations for the genes, and the hit-taxon is shown in brackets.
(DOC)
