# Supplementary material for: Phylogenomics of Reichenowia parasitica, an Alphaproteobacterial Endosymbiont of the Freshwater Leech Placobdella parasitica
Source: PLoS One. 2011 Nov 23;6(11):e28192. doi: 10.1371/journal.pone.0028192 (PMC3223239; doi:10.1371/journal.pone.0028192)
Supplement: Table S1 — List of species used for subtractive scaffolding, orthologue recovery and phylogenetic analysis. Bold font denotes the non-alphaproteobacterial species. GenBank RefSeq refers to the submission inclusive of the entire genome. (DOC) [file pone.0028192.s001.doc]

**Supplementary Table 1.** **List of species used for subtractive scaffolding, orthologue recovery and phylogenetic analysis.** Bold font denotes the non-alphaproteobacterial species. GenBank RefSeq refers to the submission inclusive of the entire genome.

| **Species** | **Class** | **Family** | **GenBank RefSeq** | **Estimated Genome Size (Mbp)** |
| --- | --- | --- | --- | --- |
| *Acetobacter pasteurianus* | Alphaproteobacteria | Acetobacteraceae | NC_013209.1 | 3.328 |
| *Agrobacterium radiobacter* | Alphaproteobacteria | Rhizobiaceae | NC_011985.1 | 7.26491 |
| *Agrobacterium tumefaciens* | Alphaproteobacteria | Rhizobiaceae | NC_00306.2 | 5.66716 |
| *Agrobacterium vitis* | Alphaproteobacteria | Rhizobiaceae | NC_011989.1 | 6.33537 |
| ***Aquifex aeolicus*** | Aquificae | Aquificaceae | NC_000918.1 | 1.59079 |
| *Azorhizobium caulinodans* | Alphaproteobacteria | Xanthobacteraceae | NC_009937.1 | 5.36977 |
| ***Bacillus anthracis*** | Firmicutes | Bacillaceae | NC_007530.2 | 5.50242 |
| *Bartonella grahamii* | Alphaproteobacteria | Bartonellaceae | NC_012846.1 | 2.36933 |
| *Bartonella henselae* | Alphaproteobacteria | Bartonellaceae | NC_005956.1 | 1.93105 |
| *Bartonella quintana* | Alphaproteobacteria | Bartonellaceae | NC_005955.1 | 1.58138 |
| ***Bdellovibrio bacteriovorus*** | Deltaproteobacteria | Bdellovibrionaceae | NC_005363.1 | 3.8 |
| *Beijerinckia indica* | Alphaproteobacteria | Beijerinickiaceae | NC_01058.1 | 4.41715 |
| *Bradyrhizobium japonicum* | Alphaproteobacteria | Bradyrhizobiaceae | NC_004463.1 | 9.1 |
| *Brucella abortus* | Alphaproteobacteria | Brucellaceae | NC_006932.1 | 3.28645 |
| *Brucella canis* | Alphaproteobacteria | Brucellaceae | NC_010103.1 | 3.3 |
| *Brucella melitensis* | Alphaproteobacteria | Brucellaceae | NC_003317.1 | 3.27779 |
| *Brucella suis* | Alphaproteobacteria | Brucellaceae | NC_004310.3 | 3.3 |
| ***Buchnera aphidicola*** | Gammaproteobacteria | Enterobacteriaceae | NC_002528.1 | 0.655086 |
| ***Campylobacter concisus*** | Epsilonproteobacteria | Campylobacteraceae | NC_009802.1 | 2.09901 |
| ***Candidatus Carsonella rudii*** | Gammaproteobacteria | - | NC_008512.1 | 0.16 |
| ***Candidatus Sulcia muelleri*** | Bacteroidetes | - | NC_014004.1 | 0.24 |
| *Caulobacter crescentus* | Alphaproteobacteria | Caulobacteraceae | NC_002696.2 | 4 |
| ***Chromobacterium violaceum*** | Betaproteobacteria | Neisseriaceae | NC_005085.1 | 4.75108 |
| *Ehrlichia canis* | Alphaproteobacteria | Anaplasmataceae | NC_007354.1 | 1.3 |
| *Ehrlichia chaffeensis* | Alphaproteobacteria | Anaplasmataceae | NC_007799.1 | 1.17625 |
| *Erythrobacter litoralis* | Alphaproteobacteria | Erythrobacteraceae | NC_007722.1 | 3.0524 |
| *Gluconobacter oxydans* | Alphaproteobacteria | Acetobacteraceae | NC_006677.1 | 2.92021 |
| *Jannaschia sp.* | Alphaproteobacteria | Rhodobacteraceae | NC_007802.1 | 4.386 |
| *Mesorhizobium loti* | Alphaproteobacteria | Phyllobacteriaceae | NC_002678.1 | 7.5963 |
| *Methylobacterium chloromethanicum* | Alphaproteobacteria | Methylobacteriaceae | NC_011757.1 | 6.18091 |
| *Methylobacterium extorquens* | Alphaproteobacteria | Methylobacteriaceae | NC_012808.1 | 6.86846 |
| *Nitrobacter hamburgensis* | Alphaproteobacteria | Bradyrhizobiaceae | NC_007964.1 | 5 |
| *Paracoccus denitrificans* | Alphaproteobacteria | Rhodobacteraceae | NC_008686.1 | 5.23238 |
| ***Prochlorococcus marinus*** | Cyanobacteria | Prochlorococcaceae | NC_009091.1 | 1.64188 |
| *Rhizobium etli* | Alphaproteobacteria | Rhizobiaceae | NC_010994.1 | 6.44 |
| *Rhizobium leguminosarum* | Alphaproteobacteria | Rhizobiaceae | NC_008380.1 | 7.74714 |
| *Rhodobacter capsulatus* | Alphaproteobacteria | Rhodobacteraceae | NC_014034.1 | 3.83 |
| *Rhodobacter sphaeroides* | Alphaproteobacteria | Rhodobacteraceae | NC_007494.1 | 4.607 |
| *Rhodopseudomonas palustris* | Alphaproteobacteria | Bradyrhizobiaceae | NC_008435.1 | 5.5 |
| *Rickettsia conorii* | Alphaproteobacteria | Rickettsiaceae | NC_003103.1 | 1.26876 |
| *Rickettsiaprowazekii* | Alphaproteobacteria | Rickettsiaceae | NC_000963.1 | 1.1 |
| *Rickettsia ricketsii* | Alphaproteobacteria | Rickettsiaceae | NC_009882.1 | 1.25771 |
| *Rickettsia typhi* | Alphaproteobacteria | Rickettsiaceae | NC_006142.1 | 1.1115 |
| *Ruegeria pomeroyi* | Alphaproteobacteria | Rhodobacteraceae | NC_003911.11 | 4.59 |
| *Sinorhizobium fredii* | Alphaproteobacteria | Rhizobiaceae | NC_012587.1 | 6.89574 |
| *Sinorhizobium medicae* | Alphaproteobacteria | Rhizobiaceae | NC_009636.1 | 6.83636 |
| *Sinorhizobium meliloti* | Alphaproteobacteria | Rhizobiaceae | NC_003047.1 | 6.70836 |
| ***Wigglesworthia glossinida*** | Gammaproteobacteria | Enterobacteriaceae | NC_004344.2 | 0.7053 |
| *Wolbachia endosymbiont of D. melanogaster* | Alphaproteobacteria | Anaplasmataceae | NC_002978.6 | 1.26778 |
| *Zymomonas mobilis* | Alphaproteobacteria | Sphingomonadaceae | NC_006526.2 | 2.1986 |
