# Supplementary material for: Phylogenomics of Reichenowia parasitica, an Alphaproteobacterial Endosymbiont of the Freshwater Leech Placobdella parasitica
Source: PLoS One. 2011 Nov 23;6(11):e28192. doi: 10.1371/journal.pone.0028192 (PMC3223239; doi:10.1371/journal.pone.0028192)
Supplement: Table S2 — Description of BLASTn Hits Encountered using the Reichenowia parasitica Contigs as Queries Against 50 Selected Bacterial Genomes. All hits matched at 1E-5 or lower. Hit descriptions follow the GenBank annotations for the genes, and the hit-taxon is shown in brackets. (DOC) [file pone.0028192.s002.doc]

**Table S2.** **Description of BLASTn Hits Encountered using the *Reichenowia parasitica* Contigs as Queries Against 50 Selected Bacterial Genomes.** All hits matched at 1E-5 or lower. Hit descriptions follow the GenBank annotations for the genes, and the hit-taxon is shown in brackets.

| **No. of hits in *R. parasitica*** | **E-value range** | **Hit description** |
| --- | --- | --- |
| 1156 | 0-1E-5 | (2Fe-2S)-binding domain protein [Methylobacterium chloromethanicum CM4] |
| 120 | 1E-128-4E-6 | 2-hydroxychromene-2-carboxylate isomerase protein [Rhizobium etli CFN 42] |
| 1182 | 0-1E-5 | 2-oxoisovalerate dehydrogenase beta subunit [Bradyrhizobium japonicum USDA 110] |
| 1308 | 0-1E-5 | 3-demethylubiquinone-9 3-methyltransferase [Azorhizobium caulinodans ORS 571] |
| 274 | 0-9E-6 | ABC transporter membrane spanning protein (dipeptide) [Agrobacterium vitis S4] |
| 311 | 1E-136-9E6 | aliphatic sulphonate ABC transporter [Agrobacterium radiobacter K84] |
| 938 | 0-1E-5 | amidophosphoribosyltransferase [Brucella canis ATCC 23365] |
| 67 | 4E-40-1E-5 | amino-acid ABC transporter binding protein [Bartonella quintana str. Toulouse] |
| 1160 | 0-1E-5 | aminotransferase, class I [Brucella abortus bv. 1 str. 9-941] |
| 125 | 1E-155-1E-6 | antirestriction protein [Agrobacterium vitis S4] |
| 63 | 6E-33-8E-6 | apolipoprotein N-acyltransferase [Bartonella grahamii as4aup] |
| 957 | 0-1E-5 | AraC family transcriptional regulator [Brucella melitensis bv. 1 str. 16M] |
| 551 | 1E-166-9E-6 | asparagine synthetase [glutamine-hydrolyzing] [Gluconobacter oxydans 621H] |
| 1972 | 0-9E-6 | autoaggregation protein (adhering protein) [Rhizobium etli CFN 42] |
| 386 | 4E-74-1E-5 | bacteriophage tail fiber protein [Chromobacterium violaceum ATCC 12472] |
| 1039 | 0-1E-5 | branched-chain amino acid ABC transporter periplasmic branched-chain amino acid-binding protein LivK [Rhodobacter capsulatus SB 1003] |
| 34 | 0-4E-6 | cation pump membrane (nitrogen fixation)protein [Rhizobium etli CFN 42] |
| 342 | 0-1E-5 | co-chaperonin GroES [Brucella abortus bv. 1 str. 9-941] |
| 8 | 5E-20-6E-6 | copper tolerance protein [Agrobacterium vitis S4] |
| 99 | 2E-57-9E-6 | cystathionine beta-lyase [Zymomonas mobilis subsp. mobilis NCIB 11163] |
| 1 | 9E-6 | cytochrome c oxidase assembly protein [Wolbachia endosymbiont of Drosophila melanogaster] |
| 4 | 4E-31-7E-6 | cytochrome c oxidase, subunit I [Nitrobacter hamburgensis X14] |
| 138 | 1E-154-7E-6 | cytochrome O ubiquinol oxidase, subunit III protein [Rhizobium etli CFN 42] |
| 265 | 0-1E-5 | D-amino acid dehydrogenase small subunit [Brucella suis 1330] |
| 178 | 0-8E-6 | deoxyribose-phosphate aldolase/phospho-2-dehydro-3-deoxyheptonate aldolase [Rhizobium leguminosarum bv. trifolii WSM1325] |
| 314 | 0-9E-6 | endoglucanase precursor [Rhizobium sp. NGR234] |
| 871 | 1E-169-1E-5 | extracellular solute-binding protein [Beijerinckia indica subsp. indica ATCC 9039] |
| 296 | 0-1E-5 | flagellar biosynthesis protein FliR [Brucella canis ATCC 23365] |
| 267 | 0-9E-6 | flagellar biosynthesis protein FliR [Brucella melitensis bv. 1 str. 16M] |
| 2003 | 0-1E-5 | glucokinase [Agrobacterium radiobacter K84] |
| 35 | 0-5E-6 | glycosy hydrolase family protein [Mesorhizobium loti MAFF303099] |
| 91 | 2E-59-9E-6 | GntR family transcriptional regulator [Ruegeria pomeroyi DSS-3] |
| 180 | 1E-111-9E-6 | hemolysin-type calcium-binding region, RTX [Rhodobacter sphaeroides 2.4.1] |
| 1100 | 0-1E-5 | histidine ammonia-lyase [Rhodobacter sphaeroides 2.4.1] |
| 1 | 3E-6 | H-NS family DNA-binding protein [Rhodobacter sphaeroides 2.4.1] |
| 88 | 7E-77-5E-6 | hypothetical 22.1 kDa periplasmic protein [Rhizobium sp. NGR234] |
| 4 | 6E-8-3E-6 | hypothetical protein A1G_03905 [Rickettsia rickettsii str. 'Sheila Smith'] |
| 6 | 1E-16-1E-6 | hypothetical protein aq_1163 [Aquifex aeolicus VF5] |
| 123 | 1E-172-3E-6 | hypothetical protein Arad_12331 [Agrobacterium radiobacter K84] |
| 483 | 0-1E-5 | hypothetical protein Atu4528 [Agrobacterium tumefaciens str. C58] |
| 45 | 2E-32-2E-6 | hypothetical protein Atu6049 [Agrobacterium tumefaciens str. C58] |
| 90 | 7E-50-8E-6 | hypothetical protein Atu8047 [Agrobacterium tumefaciens str. C58] |
| 1654 | 0-1E-5 | hypothetical protein Atu8164 [Agrobacterium tumefaciens str. C58] |
| 1790 | 0-1E-5 | hypothetical protein Avi_2578 [Agrobacterium vitis S4] |
| 3 | 1E-144-4E-6 | hypothetical protein Avi_9567 [Agrobacterium vitis S4] |
| 36 | 4E-40-9E-6 | hypothetical protein BH11390 [Bartonella henselae str. Houston-1] |
| 31 | 1E-11-8E-6 | hypothetical protein Bind_3812 [Beijerinckia indica subsp. indica ATCC 9039] |
| 607 | 0-1E-5 | hypothetical protein ELI_07840 [Erythrobacter litoralis HTCC2594] |
| 797 | 1E-155-1E-5 | hypothetical protein Jann_4099 [Jannaschia sp. CCS1] |
| 58 | 1E-108-6E-6 | hypothetical protein MexAM1_META2p0295 [Methylobacterium extorquens AM1] |
| 1985 | 0-1E-5 | hypothetical protein mll4271 [Mesorhizobium loti MAFF303099] |
| 3 | 1E-29-3E-6 | hypothetical protein mll9560 [Mesorhizobium loti MAFF303099] |
| 1962 | 0-1E-5 | hypothetical protein NGR_c14070 [Rhizobium sp. NGR234] |
| 392 | 1E-173-9E-6 | hypothetical protein Pden_3905 [Paracoccus denitrificans PD1222] |
| 165 | 1E-78-8E-6 | hypothetical protein Pden_4702 [Paracoccus denitrificans PD1222] |
| 43 | 5E-60-2E-6 | hypothetical protein Rleg_5777 [Rhizobium leguminosarum bv. trifolii WSM1325] |
| 155 | 1E-133-5E-6 | hypothetical protein Rleg_6295 [Rhizobium leguminosarum bv. trifolii WSM1325] |
| 18 | 2E-25-8E-6 | hypothetical protein RSP_3910 [Rhodobacter sphaeroides 2.4.1] |
| 291 | 0-9E-6 | hypothetical protein SM_b20011 [Sinorhizobium meliloti 1021] |
| 1895 | 0-1E-5 | hypothetical protein Smed_0776 [Sinorhizobium medicae WSM419] |
| 948 | 0-1E-5 | hypothetical protein SPO3085 [Ruegeria pomeroyi DSS-3] |
| 83 | 1E125-5E-6 | inner-membrane translocator [Rhizobium leguminosarum bv. trifolii WSM1325] |
| 19 | 3E-16-4E-6 | iron siderophore/cobalamin ABC transporter periplasmic iron siderophore/cobalamin-binding protein [Rhodobacter capsulatus SB 1003] |
| 1 | 7E-15 | isochorismatase family protein [Bacillus anthracis str. A0248] |
| 6 | 3E-41-1E-10 | K potassium transporter [Sinorhizobium medicae WSM419] |
| 553 | 0-9E-6 | LacI family transcription regulator [Caulobacter crescentus CB15] |
| 7 | 9E-13-8E-6 | LysR family transcriptional regulator [Agrobacterium radiobacter K84] |
| 162 | 0-9E-6 | NuoK2 NADH:quinone oxidoreductase subunit 11 (chain K) [Sinorhizobium meliloti 1021] |
| 56 | 3E-25-1E-5 | oxidoreductase [Agrobacterium vitis S4] |
| 1975 | 0-1E-5 | polyhydroxyalkonate synthesis repressor, PhaR [Rhizobium leguminosarum bv. trifolii WSM1325] |
| 26 | 2E-21-1E-5 | prevent-host-death family protein [Methylobacterium chloromethanicum CM4] |
| 1167 | 0-1E-5 | putative glycohydrolase [Rhodopseudomonas palustris BisA53] |
| 116 | 1E-156-8E-6 | putative glyoxalase protein [Rhizobium etli CFN 42] |
| 1896 | 0-9E-6 | putative hydantoin racemase protein [Sinorhizobium meliloti 1021] |
| 169 | 1E-31-9E-6 | putative phosphatase [Bdellovibrio bacteriovorus HD100] |
| 122 | 1E-140-1E-5 | quinolinate synthetase complex, A subunit [Rhizobium leguminosarum bv. trifolii WSM1325] |
| 915 | 0-1E-5 | resolvase family site-specific recombinase [Brucella suis 1330] |
| 3 | 4E-7-3E-6 | ribonuclease PH [Rickettsia conorii str. Malish 7] |
| 2 | 1E-130-6E-6 | serine hydroxymethyltransferase [Rickettsia typhi str. Wilmington] |
| 2 | 2E-6-3E-6 | single-strand binding protein [Nitrobacter hamburgensis X14] |
| 192 | 0-6E-6 | sugar ABC transporter, ATP binding protein [Rhizobium etli CFN 42] |
| 710 | 0-1E-5 | TonB-dependent receptor, plug [Paracoccus denitrificans PD1222] |
| 10 | 2E-26-4E-6 | transposase [Agrobacterium vitis S4] |
| 920 | 0-9E-6 | transposase IS3/IS911 [Nitrobacter hamburgensis X14] |
| 28 | 9E-83-9E-6 | universal stress protein [Rhodobacter sphaeroides 2.4.1] |
| 253 | 1E-179-9E-6 | uracil-xanthine permease [Sinorhizobium medicae WSM419] |
| 110 | 0-9E-6 | UvrD/REP helicase [Sinorhizobium medicae WSM419] |
| 4 | 1E-19-3E-9 | virulence VirF1 protein [Rhizobium etli CFN 42] |
| 1010 | 0-9E-6 | XoxF, PQQ-linked dehydrogenase of unknown function [Methylobacterium extorquens AM1] |
